# Oscillatory Coupling Between Neural and Cardiac Rhythms

**DOI:** 10.1177/09567976241235932

**Published:** 2024-04-03

**Authors:** Kaia S. Sargent, Emily L. Martinez, Alexandra C. Reed, Anika Guha, Morgan E. Bartholomew, Caroline K. Diehl, Christine S. Chang, Sarah Salama, Tzvetan Popov, Julian F. Thayer, Gregory A. Miller, Cindy M. Yee

**Affiliations:** 1 Department of Psychology, University of California, Los Angeles; 2 Department of Psychology, University of Konstanz; 3 Department of Psychology, University of Zurich; 4 Department of Psychological Science, University of California, Irvine; 5 Department of Psychiatry and Biobehavioral Sciences, University of California, Los Angeles

**Keywords:** cognitive neuroscience, electrophysiology, human body

## Abstract

Oscillations serve a critical role in organizing biological systems. In the brain, oscillatory coupling is a fundamental mechanism of communication. The possibility that neural oscillations interact directly with slower physiological rhythms (e.g., heart rate, respiration) is largely unexplored and may have important implications for psychological functioning. Oscillations in heart rate, an aspect of heart rate variability (HRV), show remarkably robust associations with psychological health. Mather and Thayer proposed coupling between high-frequency HRV (HF-HRV) and neural oscillations as a mechanism that partially accounts for such relationships. We tested this hypothesis by measuring phase-amplitude coupling between HF-HRV and neural oscillations in 37 healthy adults at rest. Robust coupling was detected in all frequency bands. Granger causality analyses indicated stronger heart-to-brain than brain-to-heart effects in all frequency bands except gamma. These findings suggest that cardiac rhythms play a causal role in modulating neural oscillations, which may have important implications for mental health.

The brain and body operate together through the integrated activity of the central and autonomic nervous systems (CNS and ANS, respectively). These relationships are typically conceived in terms of top-down control of peripheral physiological activity by the CNS, but there is emerging evidence of ANS physiology regulating and even organizing large-scale neural activity (Raut et al., 2021). Heart rate variability (HRV) is a well-established manifestation of ANS function that has remarkably robust associations with various domains of psychological functioning. Mather and Thayer (2018) proposed that, rather than simply reflecting shared systems supporting physiological and psychological regulation, associations between HRV and psychological health may reflect a causal influence of cardiac rhythms on neural activity. The present study examined oscillatory coupling between heart rate fluctuations as an index of ANS activity and electroencephalogram (EEG) rhythms as a measure of CNS function to determine a potential physiological mechanism through which the brain and body are dynamically co-regulated.

HRV is commonly interpreted as reflecting an ability to respond flexibly and adaptively to demands of the environment (Friedman & Thayer, 1998; Porges, 2001; Thayer & Lane, 2000). High-frequency HRV (HF-HRV), which reflects 0.15-to-0.4-Hz cyclical fluctuations in heart rate associated with respiration (see Fig. 1A), provides an index of the parasympathetic contribution to cardiac activity and is predominantly mediated through the vagus nerve. Research on individual differences in HRV, and particularly HF-HRV, has shown consistent links to various domains of psychological functioning. Higher HRV is associated with better emotion regulation (Appelhans & Luecken, 2006; Beauchaine, 2001; Porges, 1991), executive functioning (Forte et al., 2019; Thayer et al., 2009), ability to cope with stress (Fabes & Eisenberg, 1997), and overall mental well-being (Beauchaine & Thayer, 2015; Shaffer et al., 2014). Intriguingly, HRV biofeedback, in which participants learn to increase HRV through real-time feedback and breathing techniques, has been reliably found to improve functioning in several of these domains, most prominently, cognitive function and emotion regulation (Lehrer & Gevirtz, 2014). These findings suggest a directional pathway between HRV and CNS function, although the mechanism of such a relationship is unknown.

**Fig. 1.**
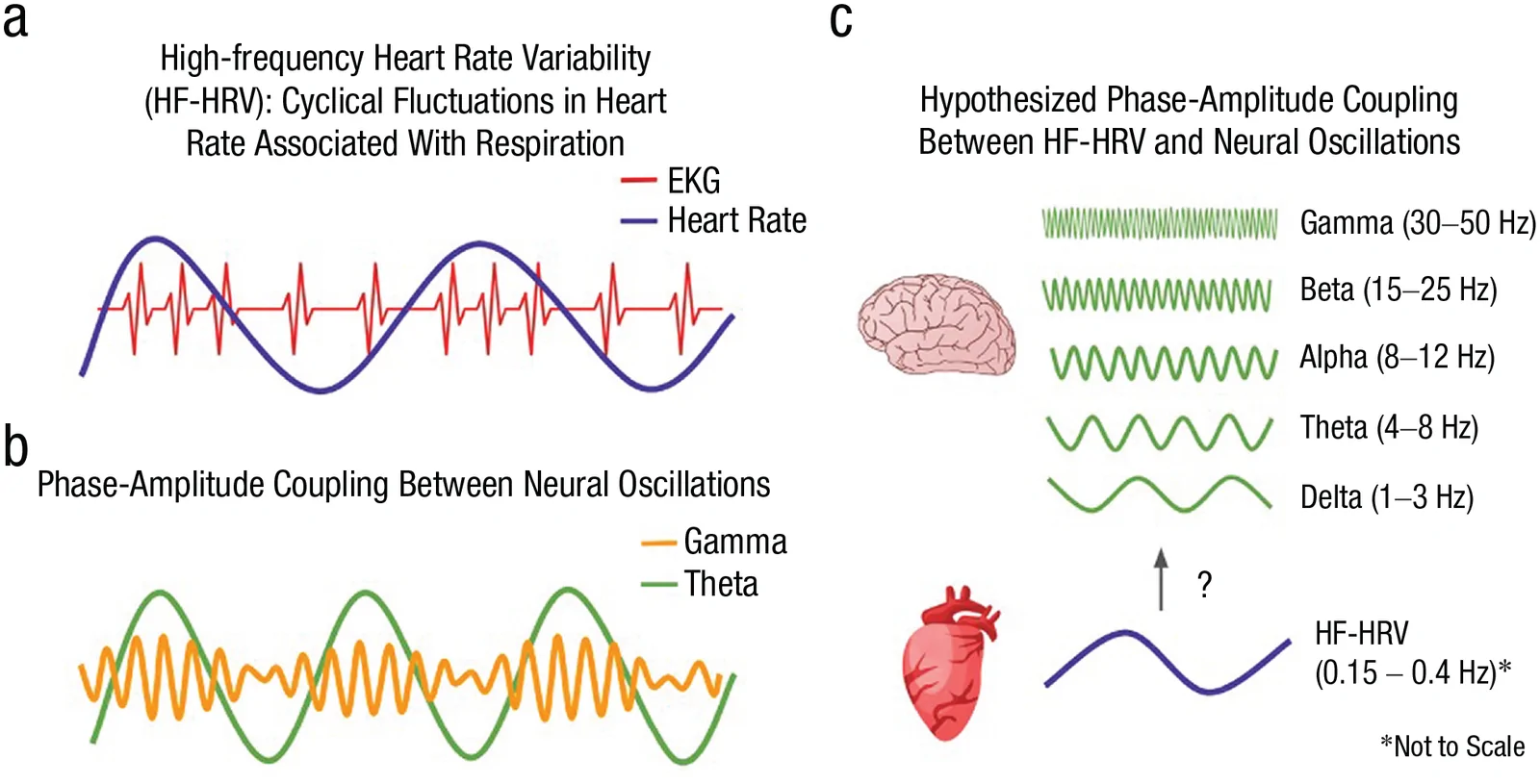
Schematic illustration of physiological oscillations and hypothesized coupling. *Oscillation* describes cyclical variation, common in physiological signals. Panel a illustrates sinusoidal increases and decreases in heart rate that correspond to the distance between peaks of the electrocardiogram. Panel b illustrates oscillatory coupling between theta and gamma oscillations, where gamma amplitude is greatest at the peak of the theta oscillation. Panel c illustrates the potential for coupling between an oscillatory pattern in heart rate (high-frequency heart rate variability) and each of five traditional electroencephalogram frequency bands.

In a recent landmark study, Raut et al. (2021) proposed a mechanism for how fluctuations in ANS activity impact large-scale spatiotemporal organization of the brain. They showed that functional magnetic resonance imaging (fMRI) signal fluctuations in humans correspond to the phase of ANS oscillations indexed by respiratory volume, HRV, and pupil size and that these signal fluctuations map onto the structure of functional neural networks. Raut and colleagues proposed that traveling waves of ANS activity contribute to the formation of functional neural networks, as they produce coherent activity in disparate brain regions that varies systematically as a function of ANS oscillatory phase. Thus, ANS oscillations appear to serve a fundamental role in organizing neural activity by modulating brainwide excitability in a spatiotemporally patterned manner.

**Statement of Relevance**

Heart rate variability (HRV), or natural increases and decreases in heart rate, is strongly associated with mental health. These associations have typically been explained by the brain’s role in “top-down” control of physiological activity. The present study tested whether cardiac rhythms might also play a “bottom-up” role in modulating neural activity, which would have important implications for understanding brain-body interactions and how they relate to psychological health. We found that electrical activity in the brain was strongly coupled to fluctuations in heart rate, with stronger heart-to-brain effects than brain-to-heart effects. These findings indicate that the link between HRV and mental health may be due in part to a causal influence of heart rhythms on brain activity. Understanding brain-body interactions as a bidirectional system can inform how we think about psychological functioning and offer new avenues for improving mental health.

Other investigators have proposed that oscillations in heart rate may foster oscillatory activity in the brain through cross-frequency coupling (e.g., Klimesch, 2018). Specifically, phase-amplitude coupling (PAC) is a mechanism by which the phase of a slow oscillation modulates the amplitude of a faster oscillation (Canolty & Knight, 2010). Although PAC has been primarily examined in neural oscillations (see Fig. 1b), several researchers have theorized that this mechanism of organization extends across physiological systems (e.g., Azzalini et al., 2019; McCraty & Zayas, 2014). Coupling has been observed between the phase of the infra-slow (~0.05 Hz) rhythm generated by the stomach and the amplitude of the EEG alpha rhythm (Richter et al., 2017), with the predominant direction of effects from the stomach to the brain. Other work has found that slow-paced breathing affects hemodynamic oscillations in the brain at 0.1 Hz (Vaschillo et al., 2016). Such findings demonstrate that oscillatory interactions previously examined only in the brain extend to brain-periphery interactions and that bottom-up visceral signals may serve a modulatory role in ongoing neural activity. Mather and Thayer (2018) proposed that PAC between heart rate oscillations and cortical activity (see Fig. 1C) may induce synchronized neural oscillations that enhance functional connectivity in brain networks supporting cognition and emotion regulation, which may also explain observed relationships between HRV and psychological functioning.

Neuroimaging studies have consistently shown that higher HRV—as either a between-subjects or a task-based difference—is associated with greater activity and connectivity of prefrontal regions (e.g., Jennings et al., 2016; Thayer et al., 2012). However, assessing interactions between HRV and neural activity on a finer temporal scale presents a distinct challenge, as measuring variability necessarily requires a defined period of time, with at least 1 min in the case of HF-HRV (Shaffer & Ginsberg, 2017). The few studies assessing time-varying relationships between HRV and EEG have relied on continuous monitoring of seizure activity in patients with epilepsy (e.g., Piper et al., 2014) or multiple-hour sleep recordings (e.g., Jurysta et al., 2003). These studies have demonstrated correlations between HRV fluctuations and EEG power, particularly in the delta band (1–4 Hz). It is unclear whether these associations are specific to pathological or sleep-related EEG changes or whether they reflect general principles of coupling between heart and brain rhythms. Furthermore, it is impossible to infer a causal relationship or physiological mechanism based on correlated time series alone.

The present study sought to determine whether heart rate oscillations modulate neural activity by assessing PAC between HF-HRV and EEG oscillations in healthy participants at rest. Despite the strong associations between HRV and psychological functioning, as well as evidence that oscillatory coupling extends across physiological systems, to our knowledge no study has explicitly tested this hypothesis. HF-HRV was modeled as a sine wave, allowing us to determine whether the amplitude of EEG oscillations in the canonical frequency bands (delta, theta, alpha, beta, and gamma) varied consistently as a function of HF-HRV phase. Granger causality analyses were performed to assess directional effects between HF-HRV phase and EEG amplitude, which we hypothesized would be stronger from HRV phase to EEG amplitude relative to the opposite direction. It was hypothesized that HRV-EEG coupling would be observed in prefrontal regions, as HRV has shown associations with prefrontal activity and connectivity in both within-subjects (Thayer et al., 2012) and between-subjects (Jennings et al., 2016) studies. Evidence of HRV phase-modulating EEG amplitude may reflect a mechanism through which ANS fluctuations influence neural activity and thus contribute to psychological functioning.

## Method

### Participants

The study included 37 participants (mean age = 21.2, *SD* = 3.0; 11 women; race/ethnicity: 11 White or European American, 11 Hispanic or Latinx, six Asian American, three Black or African American, six Other). Participants were recruited from the Greater Los Angeles community to participate in a larger study as healthy comparison participants. The Structured Clinical Interview for the *Diagnostic and Statistical Manual of Mental Disorders* (5th ed.), Research Version (SCID-5-RV; First et al., 2015), was used to determine eligibility for the larger study, and individuals were excluded from participation if they had a history of any schizophrenia spectrum or other psychotic disorders, current or recurrent major depression, bipolar disorder, obsessive-compulsive disorder, posttraumatic stress disorder, neurological disorders, history of a significant head injury, alcohol or substance dependence history, or alcohol or substance abuse in the past 6 months. Additionally, participants were excluded from the larger study if they had contraindications for MRI or if they were not a demographic match for the patient sample. In total, 41 participants were excluded (mean age = 21.2, *SD* = 2.0; 19 women; eight White or European American, 16 Hispanic or Latinx, eight Asian American, six Black or African American, three Other). Eighteen participants were excluded because of personal or family history of psychiatric or neurological disorder, five participants were excluded because of MRI contraindications, 15 participants elected to discontinue the study before participating in any study protocols beyond the SCID, and three participants were excluded because they were not a demographic match for the patient sample for the larger study.

The University of California, Los Angeles, Institutional Review Board approved all procedures, and all 37 participants provided written informed consent. Participants were instructed to refrain from using drugs or alcohol within 24 hr of the laboratory visit and to refrain from smoking cigarettes or consuming caffeine at least 1 hr prior to the study visit. Participants were debriefed and compensated at a rate of $25 per hour at the conclusion of the session.

### Psychophysiological recording

Participants were instructed to keep their eyes open and fixate on a white cross at the center of a black screen for a 5-min resting-state recording subsequent to an auditory task. Only eyes-open resting-state data were used for analysis. Electrocardiogram (EKG) was recorded at 2000 Hz with electrodes placed symmetrically on the right and left lower ribs and a ground electrode located approximately two inches below and medial to the electrode on the side corresponding to the participant’s nondominant hand. Vertical and horizontal electrooculogram (EOG) was recorded from electrodes placed above and below each pupil and adjacent to the outer canthus of each eye and referenced to the same left mastoid electrode as EEG electrodes. EEG data were obtained using a Brain Products actiCHamp active electrode system with a 10-5 distribution of 90 scalp electrodes (including mastoids) and six EOG sites and sampled at 2000 Hz. EOG and EEG sites were referenced online to the left mastoid and rereferenced offline to average mastoids (Miller et al., 1991). Electrode impedances were kept below 25 kΩ per vendor guidance. Three-dimensional coordinates of electrode positions were recorded using a CapTrak spatial digitizer, with fiducial landmarks placed on the forehead and preauricular points to aid in spatial reconstruction.

### EKG processing

The full 5 min of continuous EKG data were processed initially using QRSTool (Allen et al., 2007). Automated beat detection was used to identify each R-wave, and trained research assistants visually inspected and corrected each time series for missed beats. The corrected interbeat interval (IBI) time series was transformed from cardiac time to real time, producing a vector of IBI values, one per millisecond, with each entry being the duration of the IBI during which that time point occurred. For example, for a 700-ms IBI, the value 700 would be stored for 700 samples (i.e., the duration of the IBI). This provided temporal alignment between the IBI and EEG time series. Autoregressive modeling was then performed for each IBI time series using Kubios software (Niskanen et al., 2004) to identify a peak in the power spectrum in the HF-HRV range (0.15–0.4 Hz) for each participant, which was subsequently used to extract sinusoidal variation in heart rate (HF-HRV) at the participant’s most prominent frequency.

### EEG processing

EEG were visually inspected for myogenic artifact and other noise by trained research assistants. After artifactual segments and noisy channels were removed from the data, independent component analysis (ICA) was conducted using the Extended Infomax algorithm implemented in EEGLAB (DeLorme & Makeig, 2004) with the principal component decomposition limited to the first 30 components after applying a 1-Hz high-pass filter.

All components were visually inspected by the first author. Artifactual components corresponding to eye blink, eye movement, and cardiac activity were identified, with consultation about any ambiguous components. The resulting ICA weights excluding artifactual components were then applied to the original dataset (without artifactual segments removed and without the 1-Hz high-pass filter) such that the temporal structure of the dataset was preserved. The data were then reconstructed and down-sampled to 1000 Hz.

### Phase-amplitude coupling

**Fig. 2.**
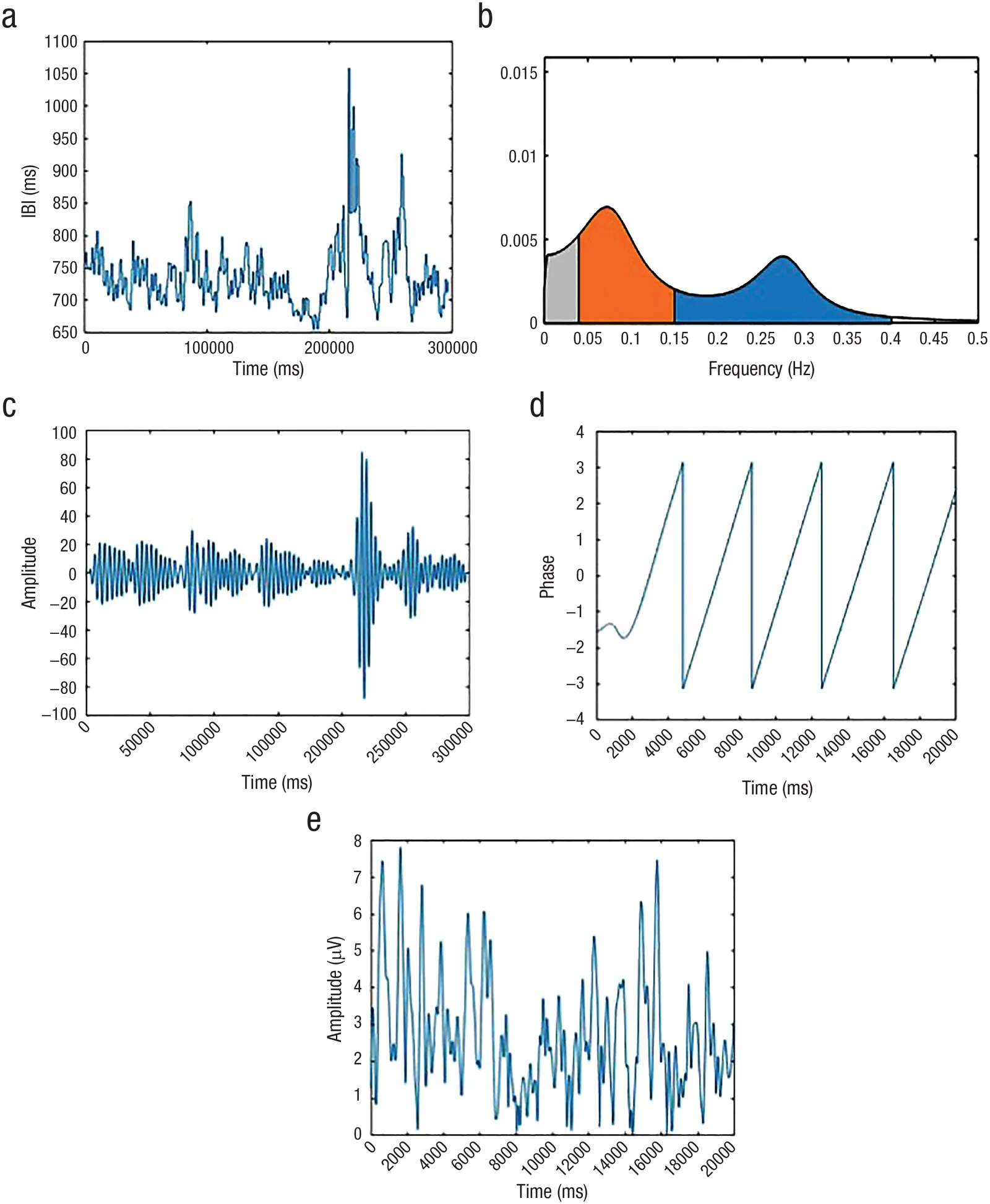
Processing steps for a representative study participant. (a) Interbeat interval (IBI) time series in real time. (b) Autoregressive power spectrum of IBI time series. Low-frequency HRV range (0.04 – 0.15 Hz) is shown in orange, and high-frequency range (0.15 – 0.4 Hz) is shown in blue. (c) IBI time series in real time, filtered at the participant’s peak high-frequency heart rate variability (HF-HRV) and visible as peak in blue segment of Figure 2b (0.273 ± 0.05 Hz). (d) Phase angle time series for HF-HRV oscillation in 20-s time window. (e) Amplitude time series for alpha oscillation in the corresponding 20-s time window.

Each participant’s real-time IBI timeseries was filtered to retain just their HF-HRV peak frequency (± 0.05 Hz) to foreground the HF-HRV oscillation. A finite impulse response (FIR) filter was designed with its order set to capture three cycles of the lower-bound frequency for each participant. The filter was applied with a Hamming window point by point over the length of the time series. The filtered time series was then Hilbert-transformed to compute a time series of phase angles for the HF-HRV oscillation. The EEG time series was averaged over select frontal channels (Fz, FCz, F1, F2, and AFz) into a single continuous time series, which was then filtered for each frequency band (delta, 1–4 Hz; theta, 4–8 Hz; alpha, 8–12 Hz; beta, 12–30 Hz; gamma, 30–50 Hz). A FIR filter with a Hamming window was applied for each frequency band, with filter order set to capture three cycles of the lower-bound frequency for each band. Filtered time series were then Hilbert-transformed to extract the amplitude envelope for each EEG frequency band. Figure 2 illustrates results of key processing steps for a representative study participant.

For each participant and each EEG band, the HF-HRV phase series and the EEG amplitude time series were used to compute a modulation index (MI) following methods developed by Tort et al. (2010). For each EEG frequency, the HF-HRV phase angles were sorted into 18 equally distributed bins spanning –pi to pi, and the average EEG amplitude was calculated for each HF-HRV phase bin. If no PAC exists between frequencies, then EEG amplitude will be distributed uniformly across HF-HRV phase bins. Deviations from a uniform distribution were quantified with the MI, with higher values (ranging between 0 and 1) indicating systematic variance in EEG power over HF-HRV phase bins and, therefore, reflecting PAC.

To assess statistical significance of PAC for each EEG frequency, a null distribution of MI values for each participant was constructed by randomly shuffling HF-HRV phase angles and recomputing MI. The percentage of randomly permuted MI values that exceeded the observed MI reflects the probability of obtaining an MI equal to or more extreme than the observed MI due to chance alone. A total of 5,000 permutations were performed, providing a *p*-value resolution of .0002.

To assess group-level statistical significance and magnitude of effects, the mean MI of the null distribution was calculated for each participant and each EEG frequency band. A Welch’s *t* test was conducted for each EEG frequency band to provide a comparison of the mean observed MI to the mean null MI at the group level. To correct for multiple comparisons given inclusion of five EEG frequency bands, a Bonferroni correction was applied with the critical alpha level adjusted to 0.05 / 5 = 0.01. Because tests of different EEG frequency bands are not independent of each other, such a Bonferroni correction is highly conservative.

To assess the direction of effects, Granger causality analyses were performed. Granger causality assesses the extent to which a time series *X* provides information that is useful in predicting future values of time series *Y*. Variable *X* is said to Granger-cause variable *Y* if values of *X* and *Y* together provide better predictions of future *Y* values than do past values of *Y* alone. In the present study, Granger analyses were performed using methods similar to those described in Munia and Aviyente (2021) using the same HF-HRV phase and EEG amplitude time series for each frequency band. Welch’s *t* tests were performed using the Granger *F* statistics to compare the magnitude of heart-to-brain versus brain-to-heart effects, with the same conservative Bonferroni correction.

## Results

### Phase-amplitude coupling

**Fig. 3.**
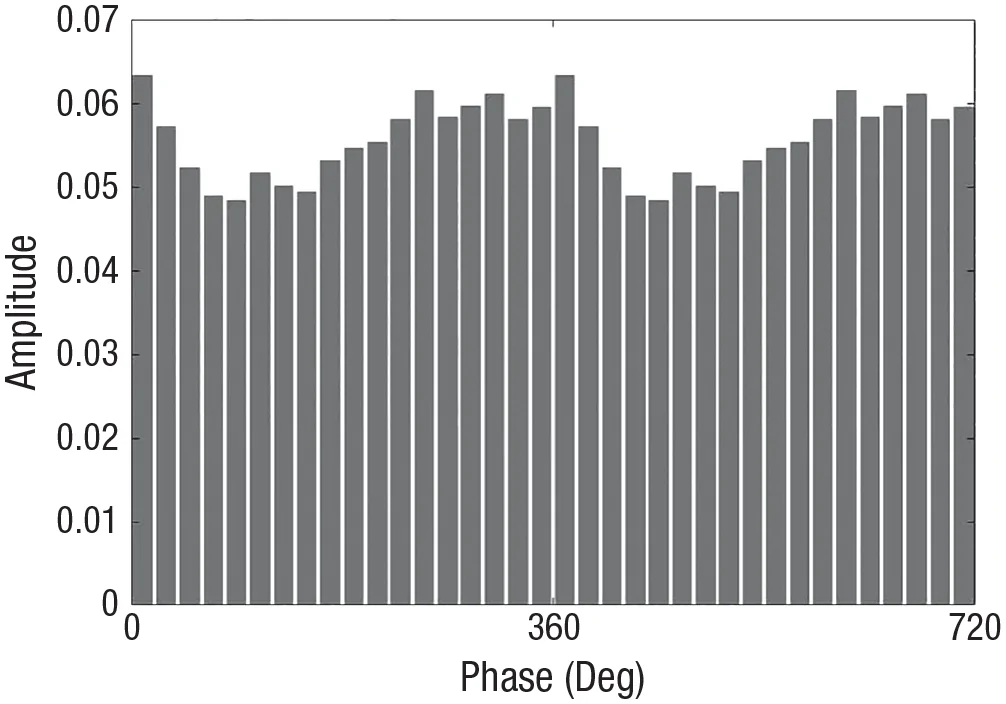
Average amplitude of the alpha oscillation in each phase bin of the high-frequency heart rate variability oscillation for a representative participant. Modulation index = 0.830e-3.

The sample showed systematic, statistically reliable variation in EEG amplitude as a function of HF-HRV phase in every frequency band. Across all participants and all EEG frequency bands, there were no permuted MI values that exceeded the observed MI (all *p* values < .0002). Figure 3 shows average alpha amplitude at each phase bin of the HF-HRV oscillation for a representative participant, illustrating that EEG alpha tracked HF-HRV phase systematically.

Table 1 provides test statistics and effect sizes. Every band showed a significant difference between group-mean observed MI values and mean null (permuted) MI values. According to standard guidelines for interpreting effect sizes (Cohen, 1992), HRV-EEG PAC showed a large effect (> 0.8) in each EEG frequency band. Alpha exhibited the largest effect, followed by delta, beta, theta, and then gamma.

**Table 1.** Test Statistics and Effect Sizes Comparing Mean Observed Modulation Index (MI) to Mean Null MI for Each Electroencephalogram Frequency Band

Frequency band	Mean observed MI ×10^–3^ (*SD*)	Mean permuted MI ×10^–3^ (*SD*)	*t* (*df* = 37)	*p*	Cohen’s *d*
Delta (1–4 Hz)	0.747 (0.423)	0.004 (0.001)	10.83	5.02e-13	2.48
Theta (4–8 Hz)	0.832 (0.600)	0.004 (0.001)	8.51	3.12e-10	1.95
Alpha (8–12 Hz)	0.681 (0.349)	0.004 (0.001)	11.93	3.01e-14	2.73
Beta (12–30 Hz)	0.313 (0.201)	0.003 (0.001)	9.51	1.76e-11	2.18
Gamma (30–50 Hz)	0.648 (0.756)	0.006 (0.007)	5.23	6.81e-06	1.20

It can be noted that the relative order of the effect sizes among the frequency bands differs from the relative order of the MI values. The seemingly discrepant pattern of results reflects differences in MI standard deviations across bands (e.g., theta has the largest MI value but also the largest standard deviation, thus a smaller effect size).

### Granger causality

To assess directional effects between HF-HRV and EEG oscillations, Granger causality analyses were performed using the same phase and amplitude time series that were used to compute MI. In each frequency band except gamma, the majority of study participants showed a significant heart-to-brain effect, whereas few showed a significant brain-to-heart effect. To evaluate the relative strength of effects in each direction, Granger *F* statistics were log-transformed to normalize the distributions and compared (heart to brain vs. brain to heart) using Welch’s *t* tests for each EEG frequency band. Table 2 shows the number of participants exhibiting significant Granger causality in each direction. Across all EEG frequency bands except gamma, the group average heart-to-brain effect was significantly stronger than the brain-to-heart effect.

**Table 2.** Number of Study Participants Showing Significant Granger Causality in Heart-to-Brain and Brain-to-Heart Directions in Each Electroencephalogram Frequency Band

Frequency band	*N* with sig. heart-to-brain	*N* with sig. brain-to-heart	Mean heart-to-brain *F* stat (*SD*)	Mean brain-to-heart *F* stat (*SD*)	*t*	*p*	Cohen’s *d*
Delta	21	3	33.41 (55.12)	1.21 (1.56)	6.10	5.68e-08	0.83
Theta	29	3	36.68 (39.05)	1.56 (1.51)	7.60	2.47e-10	1.27
Alpha	23	0	26.13 (43.93)	0.76 (0.97)	7.01	1.25e-09	0.82
Beta	7	4	3.27 (5.60)	1.23 (1.67)	2.76	0.0075	0.49
Gamma	2	0	1.30 (1.59)	0.90 (0.88)	0.44	0.66	0.31

Note: *T* statistics compare mean Granger *F* statistics in heart-to-brain versus brain-to-heart direction in each electroencephalogram frequency band. Degrees of freedom for the five bands ranged from 59.63 to 71.99.

To avoid false detections of causal interactions, we performed the same Granger analyses using time-reversed signals. This procedure is based on the rationale of Haufe and colleagues (2012), who argued that if temporal order is necessary to distinguish cause and effect, directed information flow should be reduced if temporal order is reversed. Granger estimates using the time-reversed signals were indeed consistently lower, with no *F* statistics exceeding the critical value.

Given that the EEG signal is comprised of both oscillatory and aperiodic (nonoscillatory) activity, we conducted additional analyses to provide evidence that the aforementioned results were attributable to oscillatory activity in each frequency band. We submitted each subject’s EEG power spectrum to specparam (Donoghue et al., 2020; Ostlund et al., 2022) to remove aperiodic (1/*f* ) activity and identify oscillatory peaks in the residualized power spectrum. For each subject, we identified the maximal value in each EEG frequency band and used the corresponding frequency to filter each subject’s EEG data at the subject’s peak frequency for each band. We then repeated all PAC and Granger analyses described earlier. These analyses replicated the aforementioned findings with only trivial differences, and results are reported in Supplementary Materials.

### Exploratory analyses to examine scalp topography of Granger effects

**Fig. 4.**
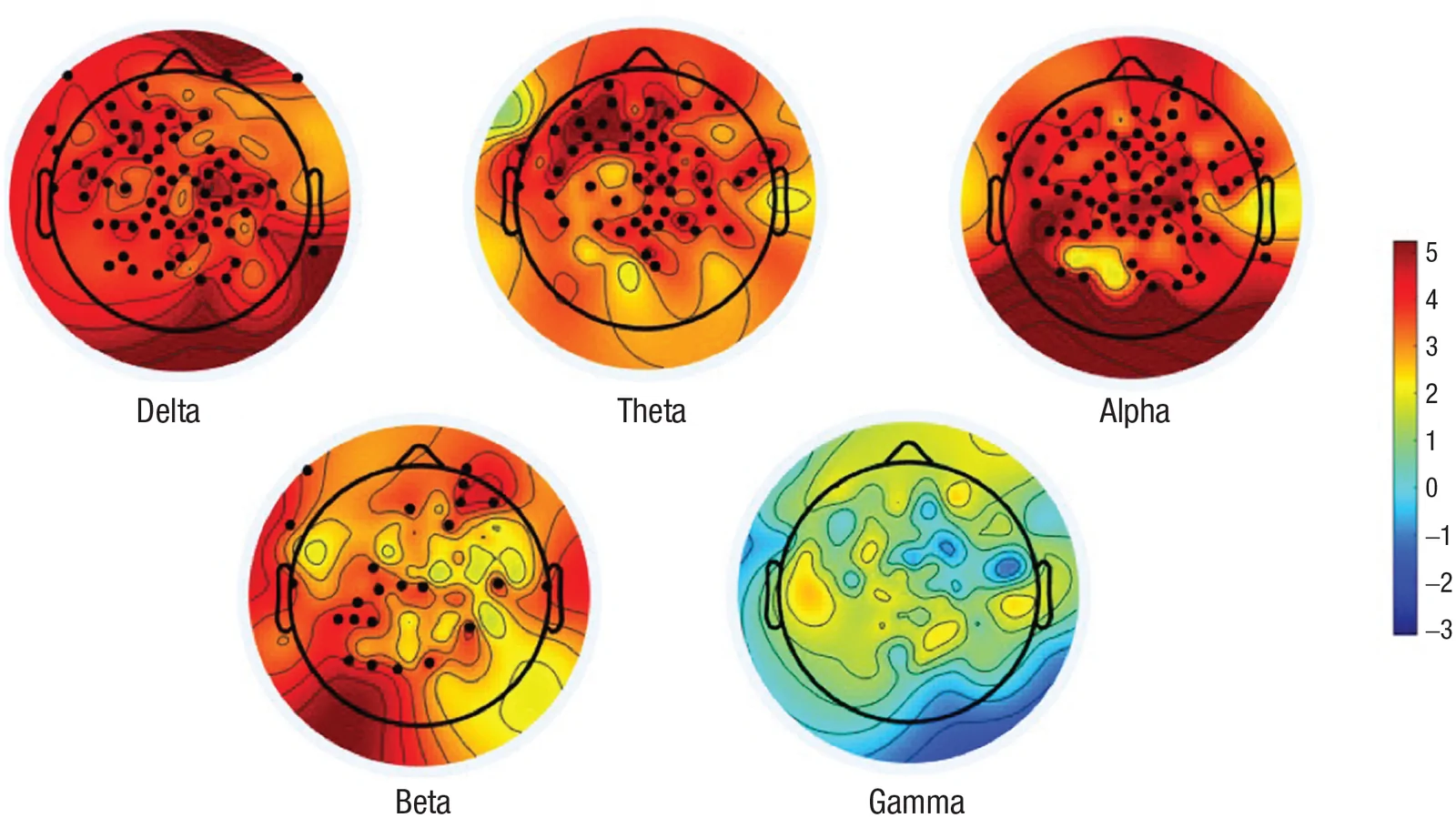
Topographic distribution of *t* statistics comparing magnitude of effects (Granger *F* statistics) in heart-to-brain versus brain-to-heart direction. Color bar reflects *t* statistics, with warmer colors indicating stronger heart-to-brain relative to brain-to-heart effects. Electrode sites that show a significant difference in heart-to-brain versus brain-to-heart effects are depicted in black.

Analyses reported thus far relied on EEG amplitude computed over frontal channels. Apart from showing HRV-EEG oscillatory coupling and the direction of the effect, it is unclear if the observed associations extend to other cortical regions of the brain. On an exploratory basis, we examined whether the scalp topography of heart-to-brain versus brain-to-heart coupling effects were frontally specific or distributed over the scalp. Using a strategy similar to that described earlier, Granger causality was examined at every scalp channel. This resulted in topographic maps of *t* statistics illustrating the relative magnitude of heart-to-brain versus brain-to-heart effects in each EEG frequency band, as shown in Figure 4. Channels that showed a significantly stronger heart-to-brain effect than brain-to-heart effect are depicted in black. As EEG provides approximately 40 degrees of freedom at most (Taulu & Larson, 2021), a Bonferroni correction with critical alpha adjusted to 0.05 / 40 = 0.00125 was used. (Because observations at each channel are not independent of each other, an unadjusted Bonferroni correction would be extraordinarily conservative.) In all frequency bands except gamma, the majority of channels showed a stronger heart-to-brain versus brain-to-heart effect, with effects at most sites far exceeding statistical significance.

## Discussion

Present findings validate the prediction of Mather and Thayer (2018) by demonstrating robust coupling between phase of the HF-HRV oscillation and amplitude of neural oscillations, with stronger evidence of Granger causality in the heart-to-brain direction than in the brain-to-heart direction across the scalp in all EEG frequency bands except gamma. These results provide evidence for oscillatory coupling between heart and brain rhythms in which cardiac activity plays a directional role in modulating neural oscillations. Such oscillatory coupling may reflect a physiological mechanism through which the ANS and CNS are dynamically integrated and potentially account for robust relationships between HRV and various indices of psychological health.

### Neural entrainment to peripheral physiological rhythms

To our knowledge, CNS entrainment to heart rate oscillations has not been previously reported, which is surprising given that entrainment is observed with other physiological rhythms (Azzalini et al., 2019; Lakatos et al., 2019). Fluctuations in alpha oscillations have been found to vary with the phase of gastric rhythms (Richter et al., 2017). Neural entrainment to respiration has also been studied extensively in the rodent brain (e.g., Adrian, 1942; Tort et al., 2018) and more recently in humans (Herrero et al., 2018; Zelano et al., 2016). Given that HF-HRV is intrinsically related to respiration, HRV-EEG coupling may be related to respiratory-EEG coupling. Disentangling these virtually inseparable processes may not be possible, and instead, cardiac and respiratory activity can be thought of as distinct but highly coupled oscillators exhibiting bidirectional relationships. For example, cardiovascular activity modulates ventilatory patterns in addition to respiration affecting heart rate, as inhalation tends to start at a preferred latency after the last heartbeat in exhalation (Dick & Morris, 2004). Therefore, HRV-EEG coupling is best considered within a broader perspective of multiple interacting oscillators in the brain and body.

Considerable research has been conducted on EEG changes in response to individual heartbeats by measuring event-related brain potentials time-locked to EKG R-waves, known as “heartbeat-evoked potentials” (HEPs; Schandry et al., 1986). HEP amplitude correlates with HRV and is similarly thought to reflect vagal afferent functioning (Huang et al., 2018; MacKinnon et al., 2013). Although less research has examined oscillatory EEG changes following heartbeats, there is evidence that heartbeats induce a phase reset of neural oscillations that may contribute to HEP generation (Lechinger et al., 2015; Park et al., 2018). It is possible that more frequent heartbeats (higher heart rate) increase the amplitude of EEG rhythms by phase-synchronizing ongoing oscillatory activity.

Slower physiological oscillations could help regulate cortical excitability and serve a role in coordinating the synchronized rhythmic activity of neural assemblies. Such organization is critical for efficient neural communication and cognitive function. The robust associations between HRV and various measures of psychological health may be substantially due to the role of peripheral physiological activity in regulating CNS excitability to promote effective neural communication. HRV-EEG coupling observed in the present study might also serve as a means of coordinating peripheral and central neural states to respond flexibly and adaptively to the environment as a single, integrated system.

### Directional effects between cardiac and neural activity

Several previous studies have employed Granger causality or similar methods to examine directional interactions between EEG and heart rate, raw EKG, or HRV power time series. Contrary to results of the present study, the majority of these studies have reported stronger brain-to-heart than heart-to-brain effects (Abdalbari et al., 2022; Greco et al., 2019; Lin et al., 2016; Pardo-Rodriguez et al., 2021). However, one study showed that HF-HRV power changes preceded changes in the delta band during sleep (Jurysta et al., 2003), and another showed stronger heart-to-brain interactions during an emotion elicitation paradigm (Candia-Rivera et al., 2022). The discrepant results may be explained by differing experimental paradigms and methods; our study is unique in that it employed phase measures of the HF-HRV oscillation.

On the basis of present and other findings, it is apparent that heart-brain interactions are bidirectional and dynamic, with multiple interacting feedback loops and mechanisms. The vagus nerve comprises both afferent (80%) and efferent (20%) connections between the brain and heart, allowing for complex feedback and feedforward connections that can be understood through a dynamical systems framework (e.g., Thayer & Lane, 2000). The present study foregrounds PAC as one mechanism through which cardiac rhythms exert a directional, modulatory influence over neural activity within a complex dynamic system.

## Limitations

It is difficult to determine whether cardiac activity exerts a direct, causal influence over neural activity via rhythmic entrainment or whether HF-HRV serves as an indirect measure of ANS fluctuations that are associated with corresponding changes in the global EEG signal. The two possibilities are not mutually exclusive, and both may contribute to HRV-EEG coupling. Furthermore, it is difficult to definitively identify directionality in PAC, particularly in relationships involving vastly different time scales. The development of mathematical techniques to model nonlinear and embedded relationships across time scales will contribute to our understanding of dynamically interacting biological processes. Finally, it is possible that the lack of significant heart-to-brain effects in the gamma band is due in part to a general signal-to-noise ratio (SNR) confound (it had the smallest PAC effect size; see Table 1). However, PAC analyses and Granger analyses differed in the relative magnitude of effects across frequency bands, with alpha showing strongest PAC and theta showing strongest heart-to-brain Granger causality. If the rank order of effect sizes was driven largely by SNR, the observed pattern should be consistent across all frequency bands for PAC and Granger causality.

### Implications and future directions

The present study tested a physiological mechanism that could account for robust relationships between HRV and psychological health. Present results support Mather and Thayer’s (2018) proposal that high-amplitude oscillations in heart rate induce oscillatory activity in the brain, which may contribute to functional connectivity in networks supporting emotion regulation and cognitive function. Determining whether and how HRV-EEG coupling modulates functional connectivity and potentially contributes to psychological health will be important next steps for future research. Such evidence would indicate that HRV-EEG coupling is a promising phenomenon on which to base development of novel, physiologically grounded approaches for improving mental health.

Oscillatory coupling between heart rhythms and brain rhythms may also have implications for conceptualizing cognition and emotion. Cognitive neuroscience has tended to conceive of regulatory processes in terms of top-down control. Present findings support the role of reciprocal and bottom-up peripheral physiological regulation of central neural activity that may support psychological phenomena. Future research may benefit from incorporating more nuanced, dynamic, and comprehensive models of reciprocal regulatory processes in the brain and body.

## Supplemental Material

sj-docx-1-pss-10.1177_09567976241235932 – Supplemental material for Oscillatory Coupling Between Neural and Cardiac Rhythms Supplemental material, sj-docx-1-pss-10.1177_09567976241235932 for Oscillatory Coupling Between Neural and Cardiac Rhythms by Kaia S. Sargent, Emily L. Martinez, Alexandra C. Reed, Anika Guha, Morgan E. Bartholomew, Caroline K. Diehl, Christine S. Chang, Sarah Salama, Julian F. Thayer, Gregory A. Miller and Cindy M. Yee in Psychological Science

## Transparency

*Action Editor:* Daniela Schiller

*Editor:* Patricia J. Bauer

*Author Contributions*

**Kaia S. Sargent:** Conceptualization; Data curation; Formal analysis; Investigation; Methodology; Software; Visualization; Writing – original draft.

**Emily L. Martinez:** Data curation; Investigation; Writing – review & editing.

**Alexandra C. Reed:** Data curation; Investigation; Writing – review & editing.

**Anika Guha:** Data curation; Investigation; Writing – review & editing.

**Morgan E. Bartholomew:** Data curation; Investigation; Writing – review & editing.

**Caroline K. Diehl:** Data curation; Investigation; Writing – review & editing.

**Christine S. Chang:** Data curation; Investigation; Writing – review & editing.

**Sarah Salama:** Data curation; Investigation; Writing – review & editing.

**Tzvetan Popov:** Conceptualization; Writing – review & editing.

**Julian F. Thayer:** Conceptualization; Writing – review & editing.

**Gregory A. Miller:** Conceptualization; Data curation; Formal analysis; Funding acquisition; Investigation; Methodology; Project administration; Supervision; Writing – review & editing.

**Cindy M. Yee:** Conceptualization; Data curation; Formal analysis; Funding acquisition; Investigation; Methodology; Project administration; Supervision; Writing – review & editing.
