# Supplementary material for: Oscillatory Coupling Between Neural and Cardiac Rhythms
Source: Psychol Sci. 2024 Apr 3;35(5):517–28. doi: 10.1177/09567976241235932 (PMC13608004; doi:10.1177/09567976241235932)
Supplement: Supplementary file 1 [file sj-docx-1-pss-10.1177_09567976241235932.docx]

**Supplementary Material: Isolating Oscillatory EEG components**

The EEG oscillatory literature has long acknowledged that typical frequency-analysis methods are vulnerable to the assumption that the time-series data are oscillatory when, in fact, EEG likely includes some non-oscillatory (aperiodic) activity. Much of that activity can be characterized with a 1/frequency (or 1/f) function, reflecting a common finding that in a typical power spectrum analysis, power tends to be higher for lower frequencies. The amplitude of such activity varies across participants, experimental task conditions, and recording sites. There has been no consensus about the relative contribution of periodic and aperiodic activity or methods for investigating this question. Using a recently developed method (originally called FOOOF for “fitting oscillations & one-over f”, <https://fooof-tools.github.io/fooof/>), which the developers renamed specparam (Donoghue et al., 2020; Ostlund et al., 2022), we sought additional evidence that results reported in the main text can be attributed primarily to oscillatory components of the EEG signal. The specparam approach attempts to model and separate the 1/f activity in a power spectrum and then to identify peaks in the power spectrum that reflect true oscillatory activity.

We applied the specparam procedure to each participant’s EEG power spectrum to identify the frequency with the maximal value in each frequency band reflecting an oscillatory peak. We then used that frequency to filter each subject’s EEG time series at the peak frequency for each band. All PAC and Granger causality analyses described in the main text (which used power in prespecified bands, rather than individual at specific frequencies) were repeated using the participant’s peak-filtered EEG timeseries and the original cardiac phase timeseries.

**Phase-amplitude Coupling**

As in the original results, there were no permuted MI values that exceeded the observed MI across all participants and EEG frequency bands (all p-values < 0.0002). Supplementary Table 1 provides test statistics and effect sizes. Consistent with the results reported in the main text, every band showed a significant difference between group mean observed MI values and mean null (permuted) MI values. The largest effect involved alpha, followed by delta, theta, beta, and gamma.

| **Frequency Band** | **Mean Observed MI x10^-3^ (SD)** | **Mean Permuted MI x10^-3^ (SD)** | **t-statistic**  **(df = 37)** | **p-value** | **Cohen’s d** |
| --- | --- | --- | --- | --- | --- |
| Delta (1-4 Hz) | 0.658 (0.511) | 0.004 (0.002) | 7.89 | 1.93e-09 | 1.81 |
| Theta (4-8 Hz) | 0.865 (0.702) | 0.004 (0.002) | 7.59 | 4.63e-09 | 1.74 |
| Alpha (8-12 Hz) | 0.647 (0.383) | 0.004 (0.002) | 10.34 | 1.81e-12 | 2.37 |
| Beta (12-30 Hz) | 0.414 (0.438) | 0.003 (0.005) | 5.77 | 1.28e-06 | 1.32 |
| Gamma (30-50 Hz) | 0.537 (0.676) | 0.006 (0.007) | 4.84 | 2.32e-05 | 1.11 |

Supplementary Table 1. Test statistics and effect sizes comparing mean observed modulation index to mean null modulation index for each EEG frequency band. Effect size based on Cohen (1992).

**Granger Causality**

In contrast to results reported in the main manuscript in which traditionally defined frequency band limits were used to compute EEG timeseries, the following reports results of Granger causality analyses performed using EEG timeseries that were filtered for each participant’s peak frequency. Similar to the original results (with the exception of beta), most participants showed a heart-to-brain effect, while few showed a significant brain-to-heart effect. Aligned with the analysis approach described in the main text, we performed the same Granger analyses using time-reversed signals to address the potential for false detection of causal interactions. Granger estimates using the time-reversed signals were consistently lower, with no F-statistics exceeding the critical value. To evaluate the relative strength of effects in the heart-to-brain and brain-to-heart directions, Granger F-statistics were log-transformed to normalize the distributions and compared (heart-to-brain vs. brain-to-heart) using Welch’s t-tests for each EEG frequency band. Supplementary Table 2 shows the number of participants exhibiting significant Granger causality in each direction. In delta, theta, and alpha, the group average heart-to-brain effect was again stronger than the brain-to-heart effect beyond the conventional .05 threshold.

| **Frequency Band** | ***N* with sig. heart-to-brain** | ***N* with sig. brain-to-heart** | **Mean heart-to-brain**  **F-stat (SD)** | **Mean brain-to-heart**  **F-stat (SD)** | **t-statistic** | **p-value** | **Cohen’s d** |
| --- | --- | --- | --- | --- | --- | --- | --- |
| Delta | 16 | 3 | 83.01 (168.18) | 1.18 (1.87) | 6.20 | 3.45e-08 | 0.69 |
| Theta | 25 | 2 | 30.65 (47.39) | 1.50 (1.57) | 5.36 | 1.13e-06 | 0.87 |
| Alpha | 18 | 0 | 15.55 (31.13) | 0.77 (0.82) | 4.94 | 5.16e-06 | 0.67 |
| Beta | 4 | 1 | 2.88 (6.48) | 1.03 (1.09) | 0.93 | 0.36 | 0.39 |
| Gamma | 1 | 0 | 1.30 (1.90) | 0.88 (0.84) | -0.22 | 0.83 | 0.29 |

Supplementary Table 2. Number of study participants showing significant Granger causality in heart-to-brain and brain-to-heart directions in each EEG frequency band using each participant’s peak frequency. t-statistics compare mean Granger F-statistics in heart-to-brain vs. brain-to-heart direction in each EEG frequency band.
